# Reduced preoperative serum choline esterase levels and fecal peritoneal contamination as potential predictors for the leakage of intestinal sutures after source control in secondary peritonitis

**DOI:** 10.1186/s13017-024-00550-x

**Published:** 2024-06-05

**Authors:** A. L. Amati, R. Ebert, L. Maier, A. K. Panah, T. Schwandner, M. Sander, M. Reichert, V. Grau, S. Petzoldt, A. Hecker

**Affiliations:** 1 Department of General, Visceral, Thoracic and Transplant Surgery, University Hospital of Giessen, Justus-Liebig University Giessen, Rudolf-Buchheim-Strasse 7, 35392 Giessen, Germany; 2 Department of General, Visceral and Transplant Surgery, University Hospital of Heidelberg, Im Neuenheimer Feld 420, 69120 Heidelberg, Germany; 3 Department of General and Visceral Surgery, Asklepios Clinic Lich, Goethestrasse 4, 35423 Lich, Germany; 4 Department of Anesthesiology, Intensive Care Medicine and Pain Therapy, University Hospital of Giessen, Justus-Liebig University Giessen, Rudolf-Buchheim-Strasse 7, 35392 Giessen, Germany

**Keywords:** Choline esterase, Suture leakage, Secondary peritonitis, Emergency laparotomy

## Abstract

**Background:**

The high rate of stoma placement during emergency laparotomy for secondary peritonitis is a paradigm in need of change in the current fast-track surgical setting. Despite growing evidence for the feasibility of primary bowel reconstruction in a peritonitic environment, little data substantiate a surgeons’ choice between a stoma and an anastomosis. The aim of this retrospective analysis is to identify pre- and intraoperative parameters that predict the leakage risk for enteric sutures placed during source control surgery (SCS) for secondary peritonitis.

**Methods:**

Between January 2014 and December 2020, 497 patients underwent SCS for secondary peritonitis, of whom 187 received a primary reconstruction of the lower gastro-intestinal tract without a diverting stoma. In 47 (25.1%) patients postoperative leakage of the enteric sutures was directly confirmed during revision surgery or by computed tomography. Quantifiable predictors of intestinal suture outcome were detected by multivariate analysis.

**Results:**

Length of intensive care, in-hospital mortality and failure of release to the initial home environment were significantly higher in patients with enteric suture leakage following SCS compared to patients with intact anastomoses (*p* < 0.0001, *p* = 0.0026 and *p* =0.0009, respectively). Reduced serum choline esterase (sCHE) levels and a high extent of peritonitis were identified as independent risk factors for insufficiency of enteric sutures placed during emergency laparotomy.

**Conclusions:**

A preoperative sCHE < 4.5 kU/L and generalized fecal peritonitis associate with a significantly higher incidence of enteric suture insufficiency after primary reconstruction of the lower gastro-intestinal tract in a peritonitic abdomen. These parameters may guide surgeons when choosing the optimal surgical procedure in the emergency setting.

## Background

Emergency laparotomy for secondary peritonitis is still associated with high mortality rates, albeit a substantial decrease was registered over the last decades as a result of improved perioperative care. By implementing evidence-based guidelines, as propagated by the Surving Sepsis Campaign (SSC), more patients with abdominal sepsis survive an otherwise fatal affliction [1–4]. The adverse effect of this development is an increase in morbidity following prolonged stays on intensive care units (ICUs), leading to debilitating chronical illness, poor clinical outcomes and poor quality of life [2, 5]. There remains an evident need to further optimize emergency care delivery.

Adherence to the SSC recommendations benefits septic patients through the implementation of screening tools such as the SIRS (Systemic Inflammatory Response Syndrome) or MEWS (Modified Early Warning Score) scores for expediting diagnosis and through commitment to early treatment goals summarized in time-framed bundles [1, 3, 4, 6]. Whilst scores for early sepsis recognition, prompt treatment initiation and post-operative intensive care protocols have thoroughly been investigated, there is little evidence-based guidance for the decision-making process during source control surgery (SCS) [6–9]. Key surgical decisions that highly impact patient outcome, such as anastomosis vs. stoma placement in a peritonitic abdomen are mostly based on the surgeon’s experience and appreciation of the patients’ severity of illness.

The consideration of primary anastomosis during SCS for secondary peritonitis is fairly recent, as for many years the choice, including that of experienced surgeons, was to avoid bowel reconstruction and place stomata instead. An enterostomy negatively affects its carriers both on a physical and psychosocial level [10, 11]. While bound to a life-time risk of stoma-related complications, less than 50% of enterostomy-carriers undergo subsequent restoration of bowel continuity, a procedure with inherent morbidity [10, 12, 13]. The tendency for enterostomy creation persists even in countries with well-developed public health-care systems. According to large-scale audits and observational multicentric studies, just about one quarter of patients undergoing emergency left-sided colonic resection receive a primary anastomosis [14, 15]. This occurs despite growing evidence that in many cases primary bowel reconstruction can be safely performed, even in patients with perforated diverticulitis and purulent or fecal peritonitis [16–18]. Addressing the same issue for small bowel perforation with peritonitis, a meta-analysis concluded that there is no sufficient data to issue evidence-based recommendations of whether and when an anastomosis can safely be placed [8]. Even the recent Enhanced Recovery after Surgery (ERAS®) Society and the World Society of Emergency Surgery (WSES) guidelines for emergency laparotomy refrain from issuing detailed recommendations on the surgical approach due to lack of data or need to extrapolate from data derived from elective surgery, leaving the decision of primary anastomosis placement at the discretion of the operating surgeon [2, 7].

The surgical strategy needs of course to be tailored to the patients’ pre-existing conditions and pathophysiological response to the peritoneal contamination, ranging from compensated inflammation to septic shock, as well as to the intraoperative finding. While for elective surgery risk factors for anastomotic leakage have been identified [19, 20], and scoring systems have been developed [21], these data remain scarce in the emergency setting. Among the preoperative tumor-unrelated parameters, the systematic review by McDermott et al. found male sex, American Society of Anesthesiologists (ASA) fitness grade, renal disease, obesity, hypoalbuminemia as a marker of a poor nutritional status, and an indication for emergency surgery to significantly increase the risk of colorectal anastomotic leaks [20]. Two of the largest observational cohort studies analyzing bowel resection with or without primary reconstruction during emergency laparotomy identified fecal contamination as an independent predictor for suture leakage [14, 22]. Both patient cohorts were heterogenous with only 10–30% having peritonitis as an indication for emergency surgery. The authors acknowledged that the lack of data depicting preoperative nutritional deficits limited their risk assessment, as malnutrition has repeatedly been identified as an independent predictor of anastomotic leakage and sepsis [22–24]. Low serum albumin, a high C-reactive protein (CRP)-albumin ratio and low serum choline esterase (sCHE) as markers of malnutrition have been linked to a disturbed postoperative wound healing, including that of gastrointestinal (GI) sutures and to a poor prognosis in septic patients, highlighting the need of taking these factors into consideration when placing sutures in a septic surrounding [23–27].

Switching the focus towards the human, decision-making “surgeon factor”, the trend for on-going sub-specialization benefits patients undergoing elective oncologic surgery, but it has been shown to impair the outcomes of emergency surgery, when the required operation is not part of the surgeons’ usual procedural spectrum [28]. Nevertheless, reality confronts all general surgeons on duty regardless of experience and sub-specialization with the risk/benefit assessment of primary bowel reconstruction in secondary peritonitis. Defining patient-associated factors and factors related to the intraabdominal pathology that might facilitate this decision remains of utmost importance.

The aim of this retrospective data analysis is to identify quantifiable pre- and intraoperative parameters which might facilitate the surgeon’s decision for or against a primary bowel reconstruction in a peritonitic abdomen.

## Material and methods

### Patient selection

All consecutive patients (≥ 18 years of age) who underwent emergency laparotomy for secondary peritonitis between January 2014 and December 2020 in the Department of General, Visceral, Thoracic and Transplant Surgery of the University Hospital of Giessen were retrospectively evaluated according to the following criteria defined for inclusion or exclusion from the study.

*Inclusion criterium* The main inclusion criterium was primary bowel reconstruction through placement of intestinal sutures on the lower GI tract (below the ligament of Treitz) during emergency laparotomy for intraoperatively confirmed localized or generalized, purulent or fecal peritonitis.

*Exclusion criteria* We excluded traumatic GI perforations due to blunt or penetrating trauma. Also excluded were patients with perforated acute appendicitis and cholecystitis, as morbidity and mortality rates in these cases are known to be significantly lower than in the case of hollow viscus perforation [29, 30]. Patients with chronic and contained enteric fistulae, repaired in an elective setting were not considered for inclusion in the study. Patients undergoing exclusive repair of the upper GI tract or of insufficient pancreatico-billiary reconstructions during emergency laparotomy for confirmed peritoneal contamination were excluded. Further exclusion criteria were discontinuity resections or enterostomy placement orally from the site of primary reconstructive sutures.

All surgeries were either performed or supervised by a consultant surgeon who was primarily responsible for deciding the surgical strategy. All patients were treated according to the institutional standard of care.

### Study variables

The preoperative parameters collected from the included patients were demographics: age, gender, body mass index (BMI) and pre-existing conditions: chronic pulmonary, liver or kidney disease, history of cardiovascular disease, diabetes, previously diagnosed malignancy as well as chronic inflammatory disease. ASA classification score was calculated based on the known comorbidities at the time of SCS. Previous medication that could influence postoperative morbidity in terms of bleeding or impaired wound healing, such as anticoagulant and antiplatelet agents, immunosuppressives, or chronic steroid therapy was also registered. The preoperative laboratory parameters were chosen to depict inflammation (leukocyte count, CRP), anemia (hemoglobin), liver function (sCHE, bilirubin) and kidney function (creatinine). These parameters were part of the standard blood analysis panel for surgical emergencies. SCHE was determined in the hospital’s central laboratory by the means of ultraviolet–visible (UV–VIS) spectrophotometry using the ADVIA assay kit from Siemens Healthineers (Erlangen, Germany).

The collected intraoperative data included the condition identified during SCS as the cause or main contributor to the disruption of bowel integrity such as mesenteric ischemia, mechanical bowel obstruction, or bowel inflammation. Furthermore, the location of enteric sutures placed during SCS, intraoperative blood loss and procedure time were recorded.

**Fig. 1:**
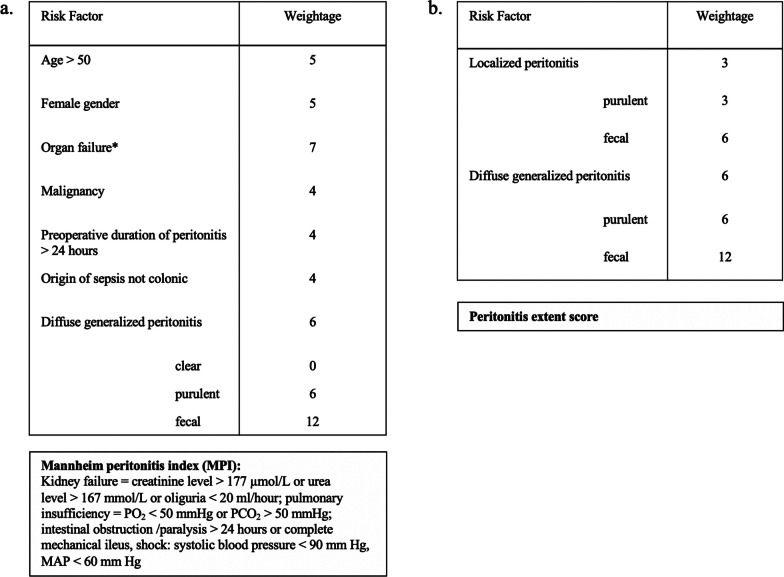
Scores for the assessment of peritonitis severity. **a** Mannheim peritonitis index (MPI); **b** peritonitis extent score used in this study. PO_2_—partial pressure of oxygen; PCO_2_—partial pressure of carbon dioxide; MAP—mean arterial pressure

We calculated the Mannheim peritonitis index (MPI), as a validated score for predicting mortality from secondary peritonitis that takes into account the 8 parameters listed below (Fig. 1a.). For the sole purpose of documenting the extent of peritoneal contamination and quality of the peritoneal exudate we developed a simplified score, leaning on the MPI that we entitled peritonitis extent score (Fig. 1b.).

### Outcome measures

The primary outcome parameter was the postoperative leakage rate of the intestinal sutures placed during SCS. Sutures were classified as insufficient either when leakage was directly confirmed during revision surgery or when computed tomography delivered strong proof of leakage, such as extra-enteric contrast medium spillage with the consequence of therapy limitation for patients deemed too critical for revision surgery. A secondary outcome measure was postoperative mortality, either in-hospital or within 100 days of the procedure if discharged. Also considered was the sequential organ failure assessment (SOFA) score upon ICU admittance and on the second postoperative day as well as surgical morbidity other than suture leakage. This included postoperative bleeding and superficial as well as deep surgical site infections. ICU length of stay and in-hospital length of stay were also recorded.

### Statistical analysis

Data analysis was performed using GraphPad Prism (Version 9 for Windows, GraphPad Software, San Diego, CA, USA, www.graphpad.com). Continuous variables are presented as median and interquartile range (IQR) and were analyzed using the Mann–Whitney U test. Categorical variables are shown as numbers with percentages, n (%), and were compared using a chi-squared test or Fisher’s exact test, as appropriate. Associations between preoperative as well as intraoperative parameters and suture leakage were investigated by univariate logistic regression. Variables with statistically significant association on univariate analysis were included in a multivariable logistic regression model. The multiple logistic regression model was tested for multicollinearity by calculating the variance inflation factors (VIF) for each variable included.

Survival curves were generated using the Kaplan–Meier method and compared using a log-rank test. Spearman’s rho rank correlation was used to determine statistical dependence between preoperative parameters. Results are given as the Spearman’s rank correlation coefficient (r) and respective significances.

P values of ≤ 0.05 (two-sided) were considered statistically significant.

## Results

### Pre- and intraoperative characteristics of patients with lower GI sutures placed during SCS for secondary peritonitis

**Fig. 2:**
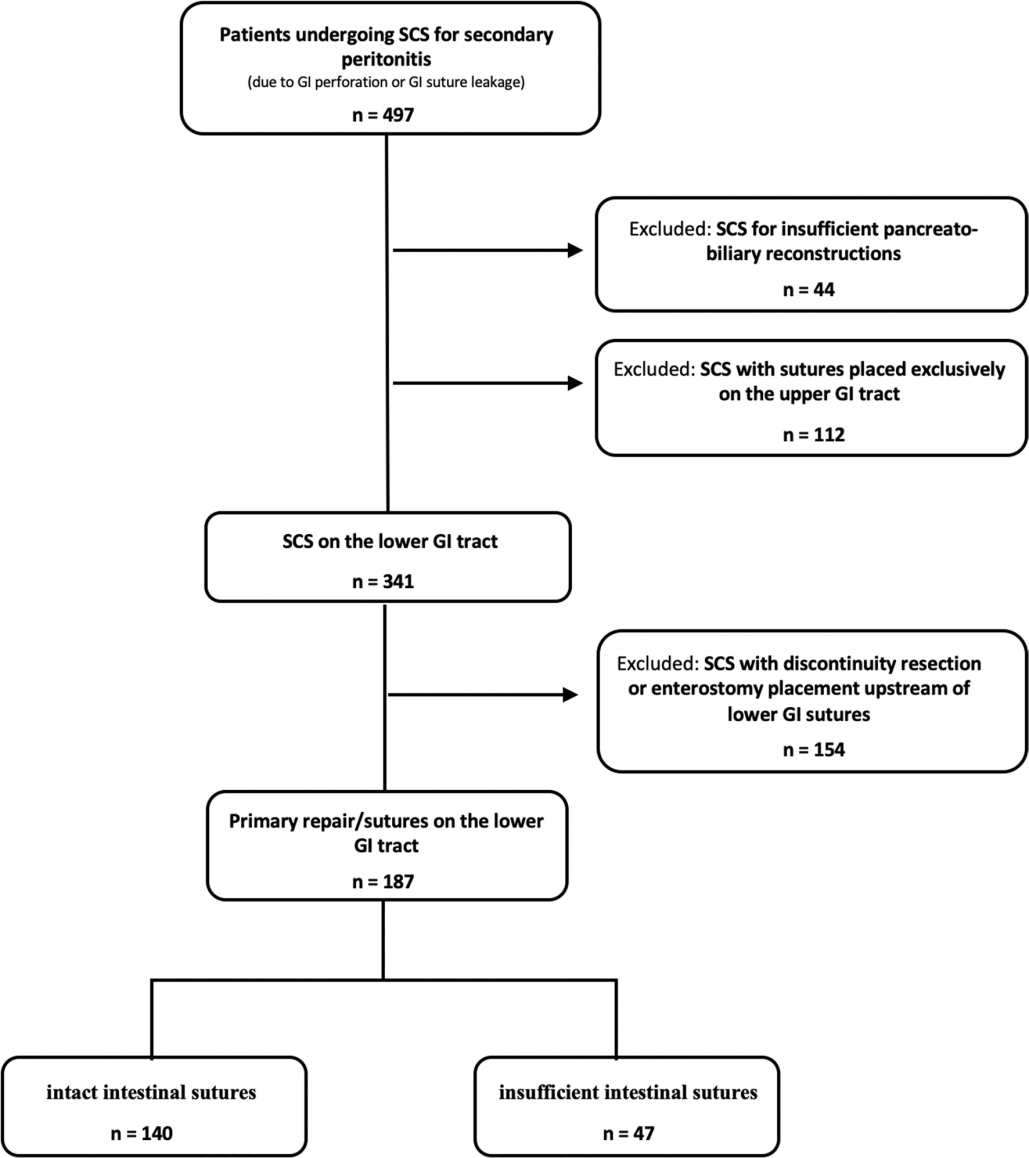
Patient selection and subgroup definition. 497 patients undergoing source control surgery (SCS) for secondary peritonitis due to gastrointestinal (GI) perforation or suture leakage were screened for study inclusion. 187 patients for whom SCS consisted of primary reconstruction of the lower GI integrity were included in the study. Two patient subgroups were defined, based on whether the enteric sutures placed during SCS remained intact (140 patients) or developed a leakage (47 patients)

A total number of 497 patients underwent SCS for secondary peritonitis caused either by hollow viscus perforation or insufficiency of electively placed GI sutures. 122 patients with source control interventions exclusively on the upper GI tract, and 44 patients with SCS consisting in the exclusive repair of insufficient pancreatico-billiary reconstructions were excluded. Of the 341 patients needing source control intervention on the lower GI tract, 154 received diverting or permanent enterostomies, leaving 187 patients with primary reconstructions of the lower GI tract during SCS for further analysis. These 187 patients were divided into two patient subgroups depending on whether the primary lower GI reconstructions performed during SCS remained intact (140 patients) or developed a leakage (47 patients) (Fig. 2).

Suture leakage was detected with a mean latency of 7.9 days from SCS and was confirmed either by revision surgery for 45 of the patients or by CT-scans showing direct extraluminal leakage of enteric contrast medium for the other two patients, whose therapy was limited prior to revision surgery due to poor overall prognosis. For the other subgroup of 140 patients the sutures placed during SCS remained intact.

There was no significant difference in basal characteristics between the two patient subgroups. The subgroup of patients with intact sutures, however, had lower CRP and bilirubin levels as well as higher sCHE activity prior to SCS compared to the patients developing suture leakage (Table 1).

**Table 1:** Preoperative characteristics of patients with suture placement on the lower gastrointestinal tract during source control surgery (SCS) for secondary peritonitis

Characteristics	Intact intestinal sutures (n = 140)	Insufficient intestinal sutures (n = 47)	*p* value
*Demographics*			
Male gender, n (%)	80 (57.1%)	31 (65.9%)	0.2871
Age, years (IQR)	61 (46–75)	63 (54–76)	0.2030
BMI, kg/m^2^ (IQR)	25.1 (22–28.4)	26.2 (23.5–29.8)	0.1439
*Underlying conditions*			
Chronic pulmonary disease, n (%)	29 (20.7%)	11 (23.4%)	0.6972
Malignancy, n (%)	51 (36.4%)	23 (48.9%)	0.1292
Chronic liver disease, n (%)	10 (7.1%)	5 (10.6%)	0.4453
Chronic kidney disease, n (%)	32 (22.8%)	12 (25.5%)	0.7084
Cardio-vascular disease, n (%)	54 (38.5%)	18 (38.3%)	0.9734
Diabetes, n (%)	27 (19.3%)	10 (21.3%)	0.7669
Chronic inflammatory disease, n (%)	21 (15.4%)	6 (12.2%)	0.5869
*Previous medication*			
Therapeutic anticoagulation, n (%)	28 (20.0%)	11 (23.4%)	0.6192
Platelet aggregation inhibitors, n (%)	43 (30.7%)	14 (29.8%)	0.9049
Chronic steroid therapy, n (%)	21 (15.0%)	4 (8.5%)	0.2580
Chemotherapy within last 3 months, n (%)	12 (8.5%)	4 (8.5%)	0.9897
Immunosuppressants, n (%)	9 (6.4%)	2 (4.2%)	0.5838
Statins, n (%)	35 (25.0%)	9 (19.1%)	0.4132
Lifestyle risk factors			
Malnutrition (BMI < 20), n (%)	19 (13.5%)	5 (10.6%)	0.6029
Obesity (BMI ≥ 30), n (%)	31 (22.1%)	15 (31.9%)	0.1783
*Preoperative laboratory parameters*			
Leukocytes, 10^9^/L (IQR)	13.55 (9.3–19.3)	10.5 (7.4–17.9)	0.0541
Hb, g/L (IQR)	106 (92–127)	104 (91–123)	0.4377
CRP, mg/L (IQR)	173.1 (64.8–252.6)	215.4 (150.9–306.7)	**0.0324**
sCHE, kU/L (IQR)	4.5 (2.8–6.2)	3.3 (1.8–4.7)	**0.0029**
Creatinine, mg/dL (IQR)	0.9 (0.7–1.4)	0.9 (0.7–1.7)	0.3875
Bilirubin, mg/dL (IQR)	0.6 (0.3–0.9)	0.8 (0.4–1.5)	**0.0281**
Platelet count, 10^9^/L (IQR)	277 (215.3–387.3)	250 (192–308)	0.0802
ASA score, (IQR)	3 (3–3)	3 (3–3)	0.4334

*IQR* interquartile range, *BMI* body mass index, *Hb* hemoglobin, *CRP* C-reactive protein, *sCHE* serum choline esterase, *ASA* American Society of Anesthesiologists

Secondary peritonitis requiring SCS was caused in similar proportions in both patient subgroups by gastrointestinal perforation (75.7% vs. 62.5%) or disruption of electively placed gastrointestinal sutures (24.3% vs. 37.5%). There was no significant difference in the location (small vs. large bowel) of the sutures placed during SCS. The length of source control procedures and intraoperative blood loss did not significantly differ in patients with intact vs. insufficient sutures (Table 2).

**Table 2:** Intraoperative characteristics of patients with suture placement on the lower gastrointestinal tract during source control surgery (SCS) for secondary peritonitis

Intraoperative characteristics	Intact intestinal sutures (n = 140)	Insufficient intestinal sutures (n = 47)	*p* value
*Cause of peritonitis*			
Perforation, n (%)	106 (75.7%)	25 (62.5%)	0.1096
Anastomotic leakage, n (%)	34 (24.3%)	15 (37.5%)	
*Underlying cause*			
Mesenteric infarction, n (%)	20 (14.3%)	9 (22.5%)	0.1259
Inflammation, n (%)	36 (25.7%)	5 (12.5%)	
Mechanical obstruction, n (%)	6 (4.3%)	3 (7.5%)	
Tumor perforation, n (%)	12 (8.5%)	0 (0%)	
Iatrogenic, n (%)	14 (10%)	4 (10%)	
Other or undetermined causes, n (%)	52 (37.1%)	19 (47.5%)	
*Mannheim peritonitis index (MPI)*			
MPI Grade I (< 21), n (%)	91 (65%)	23 (48.9%)	0.0726
MPI Grade 2 (21–29), n (%)	23 (16.4%)	8 (17.0%)	
MPI Grade 3 (> 29), n (%)	26 (18.5%)	16 (34.0%)	
*Location of enteric sutures*			
Small bowel, n (%)	70 (47.3%)	27 (50%)	0.9238
Small to large bowel, n (%)	48 (32.4%)	16 (29.6%)	
Large bowel, n (%)	30 (20.2%)	11 (20.3%)	
Intraoperative blood loss, mL (IQR)	200 (100–400)	200 (50–500)	0.2628
Operation time, minutes (IQR)	123.5 (97–169.8)	125 (88–156)	0.6644

*IQR* interquartile range, *MPI* Mannheim peritonitis index

### Postoperative outcomes of patients with lower GI sutures placed during SCS for secondary peritonitis

While SOFA scores immediately upon postoperative ICU admittance were similarly elevated in both patient subgroups, the subgroup of patients with intact enteric sutures had a significantly lower SOFA score on the second postoperative day and therefore a significant improvement in organ functionality.

Both incisional as well as intra-abdominal space infection were significantly higher in the subgroup of patients with insufficient enteric sutures. These patients also had a significantly prolonged stay on the ICU of a median of 8 days, almost three times longer than the intensive care period required by patients with intact sutures. The in-hospital mortality of 38.3% was also significantly higher in the subgroup of patients with suture leakage, of whom only 25.5% were released in their initial home environment (Table 3).

**Table 3:** Postoperative outcomes of patients with sutures placement on the lower gastrointestinal tract during source control surgery (SCS) for secondary peritonitis

Postoperative outcomes	Intact intestinal sutures (n = 140)	Insufficient intestinal sutures (n = 47)	*p* value
SOFA-score POD 0, (IQR)	5 (1–9)	5 (2–10)	0.2200
SOFA-score POD 2, (IQR)	3 (0–6)	5 (2–10)	**0.0055**
*Surgical morbidity*			
Postoperative bleeding, n (%)	7 (5.0%)	5 (10.6%)	0.1806
Incisional surgical site infection (SSI), n (%)	18 (12.8%)	19 (40.4%)	**0.0001**
Organ or space SSI, n (%)	14 (10.0%)	11 (23.4%)	**0.0262**
ICU length of stay, days (IQR)	3 (2–6)	8 (4–23)	** < 0.0001**
In-hospital length of stay, days (IQR)	14 (8.2–22.7)	29 (18–50)	** < 0.0001**
In-hospital mortality, n (%)	24 (17.1%)	18 (38.3%)	**0.0026**
Patients released to initial home environment, n (%)	75 (53.5%)	12 (25.5%)	**0.0009**

*SOFA* sequential organ failure assessment, *POD* postoperative day, *IQR* interquartile range, *SSI* surgical site infection, *ICU* intensive care unit

### Univariate and multivariate analysis of preoperative and intraoperative factors associated with leakage of lower GI sutures placed during SCS for secondary peritonitis

The following variables showed a statistically significant association with suture leakage in the univariate analysis (Table 4): preoperative CRP levels (*p* =0.0232), preoperative sCHE activity (*p* =0.0019) and the peritonitis extent score (*p* =0.0045). We chose not to include the MPI in our analysis as at least three of the parameters needed to calculate the MPI (age, sex, malignancy) showed no significant association with our primary outcome measure. Other parameters known to influence the outcome of colorectal sutures placed during elective surgery, such as BMI, chronic steroid intake and ASA-score [19, 20] were not significantly associated with the outcome (intact vs. insufficient) of sutures placed on the lower GI tract during SCS for secondary peritonitis. In the multivariate analysis sCHE activity and the peritonitis extent score remained independent predictors for suture outcome (*p* =0.0472 and *p* =0.0234, respectively).

**Table 4:** Uni- and multivariable logistic regression analyzing preoperative and intraoperative risk factors for insufficiency of intestinal sutures placed during source control surgery for secondary peritonitis

Risk factor	Univariate		Multivariate		
	OR (95% CI)	*p*	OR (95% CI)	*p*	VIF
Age, years	1.015 (− 0.004 to 0.035)	0.1371			
Sex	0.6882 (0.3390 to 1.357)	0.2837			
BMI, kg/m^2^	1.036 (0.9976 to 1.081)	0.0663			
Malignancy	1.672 (0.8561 to 3.270)	0.1311			
Chronic steroid intake	0.5271 (− 1.914 to 0.3928)	0.2383			
Hb, g/L	0.9958 (0.9823 to 1.009)	0.5392			
Bilirubin, mg/dL	1.060 (0.8215 to 1.329)	0.6199			
CRP, mg/L	1.003 (1.000 to 1.006)	**0.0232**	1.002 (0.9984–1.005)	0.3550	1.248
sCHE, kU/L	0.9998 (0.9996 to 0.9999)	**0.0019**	0.9998 (0.9996–1.000)	**0.0472**	1.159
Peritonitis extent score	1.048 (1.015 to 1.085)	**0.0045**	1.041 (1.006–1.079)	**0.0333**	1.088
ASA score	0.7932 (0.4826 to 1.287)	0.3495			

*OR* odds ratio, *CI* confidence interval, *BMI* body mass index, *Hb* hemoglobin, *CRP* C-reactive protein, *sCHE* serum choline esterase, *ASA* American Society of Anesthesiologists

### Correlation of sCHE activity with suture outcome and patient survival after SCS for secondary peritonitis

**Fig. 3:**
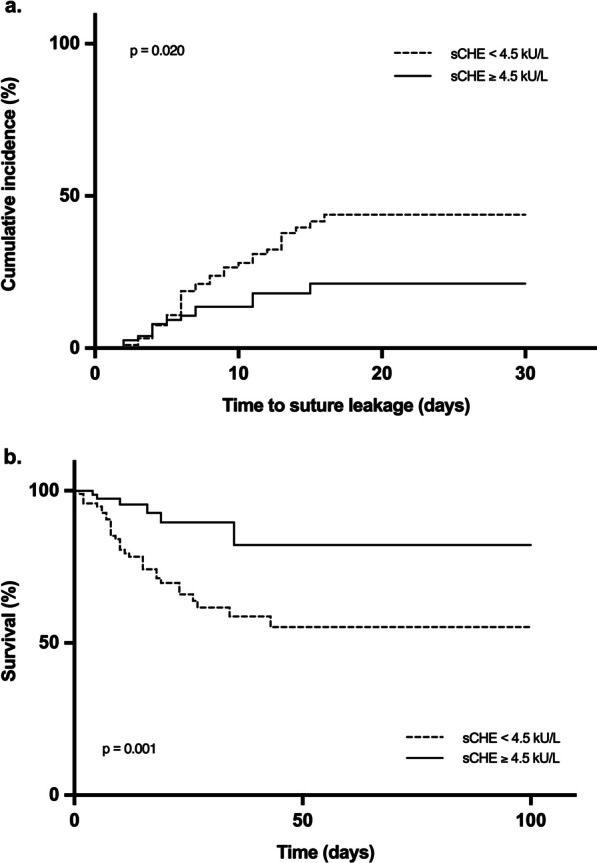
Occurrence of suture leakage and survival of patients in dependence of serum choline esterase activity (sCHE), < 4.5 kU/L vs. ≥ 4.5 kU/L. Shown is the cumulative incidence of suture leakage (**a**) and the survival (**b**) after source control surgery (SCS) for secondary peritonitis with suture placement on the lower gastrointestinal tract, in dependence of preoperative sCHE activity

We analyzed the correlation of low preoperative sCHE activity and the development of suture leakage. As a cut-off value we took the lower end of the reference interval of 4.5 kU/L. Patients with a sCHE < 4.5 kU/L (n = 96) developed a significantly higher rate of suture insufficiency (*p* =0.02) and had a significantly higher mortality (*p* =0.001) than patients with sCHE activity within the normal range (Fig. 3).

### Correlation of CRP/sCHE ratio with patient survival after SCS for secondary peritonitis, in dependence of suture outcome

**Fig. 4:**
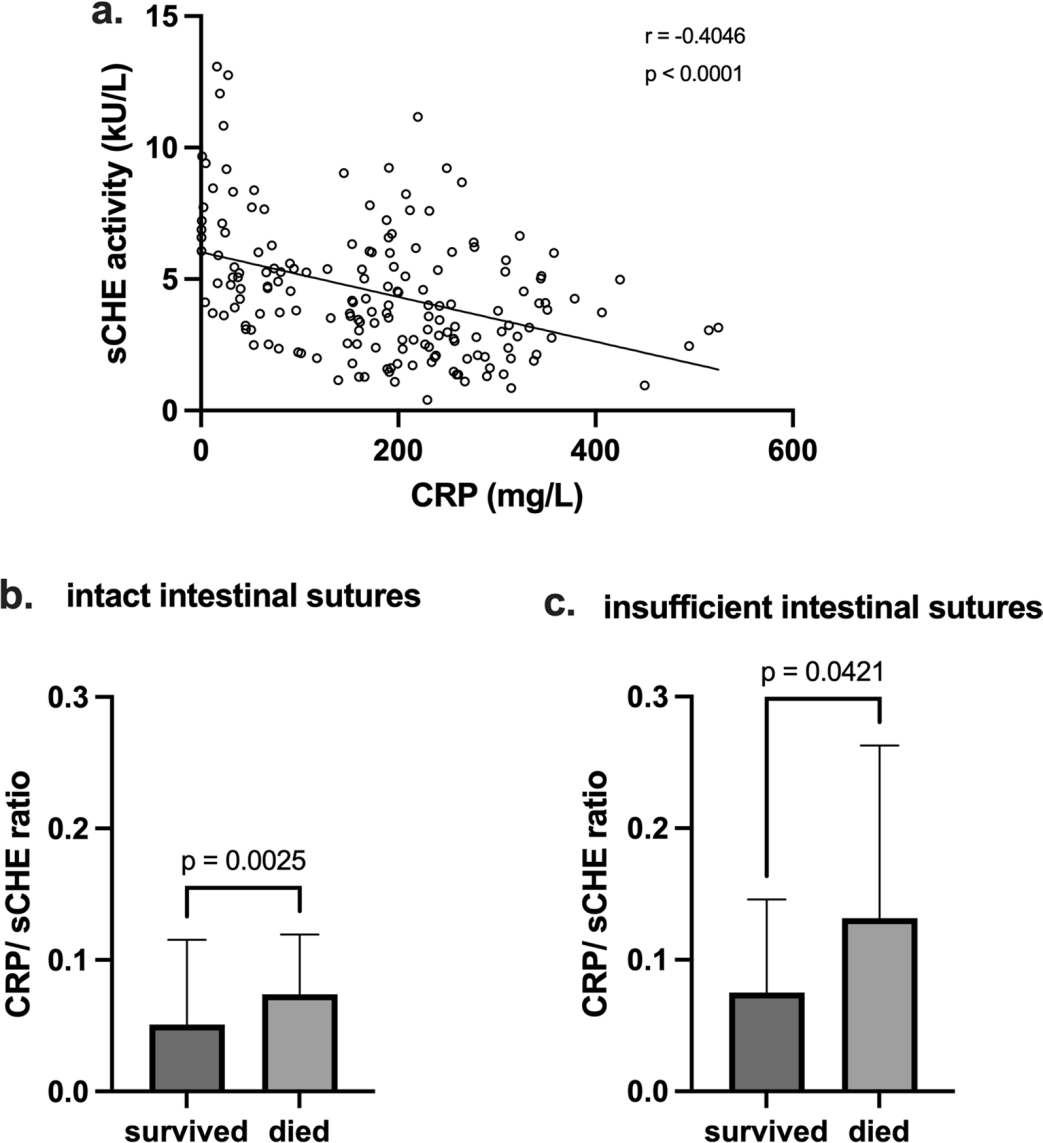
Correlation between preoperative C-reactive protein (CRP) and serum choline esterase (sCHE) (**a**) and impact of the CRP/sCHE ratio on patient mortality in dependence of enteric suture outcome: intact (**b**) versus insufficient (**c**)

No multicollinearity issue was detected in the multiple logistic regression model, since the calculated variance inflation factors (VIF) for each independent variable were below 1.5. Nevertheless, there was a negative correlation detected between preoperative CRP and sCHE activity with a Spearman correlation coefficient of − 0.4046. The CRP/sCHE ratio was able to discriminate between death and survival following SCS for secondary peritonitis in both patient subgroups with intact (*p* =0.0025) and insufficient (*p* =0.0421) enteric sutures respectively (Fig. 4).

### Correlation of peritonitis extent with suture outcome and patient survival after SCS for secondary peritonitis

**Fig. 5:**
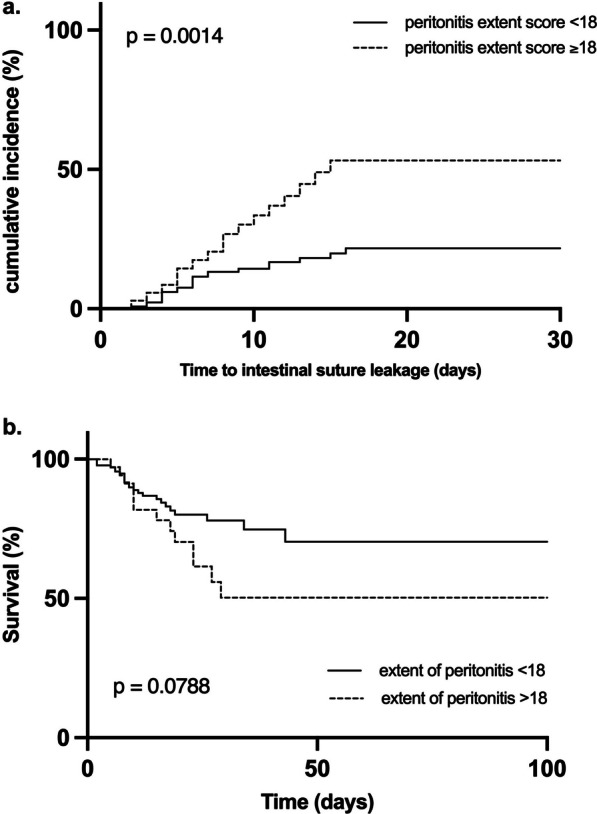
Occurrence of suture leakage and survival of patients in dependence of peritonitis extent. Shown is the cumulative incidence of suture leakage (**a**) and the survival (**b**) after source control surgery for secondary peritonitis with suture placement in the lower gastrointestinal tract, in dependence of the intraoperatively detected peritonitis extent, quantified through the peritonitis extent score (Fig. 2b in Material and Methods)

Patients with a peritonitis extent score of ≥ 18, implying a generalized fecal peritonitis, had a significantly higher incidence (*p* =0.0014) of enteric suture leakage compared to patients with a less severe degree of peritonitis. There was no significant difference but a noticeable trend (*p* =0.0788) in patient survival when taking the extent of peritoneal contamination into account (Fig. 5).

## Discussion

This study aimed to identify quantifiable preoperative and intraoperative parameters associated with a high risk of leakage for sutures placed on the lower GI tract during SCS for secondary peritonitis. These parameters could serve as an everyday tool for surgeons to decide between a primary intestinal reconstruction vs. enterostomy placement. In our patient group, 25.1% of the sutures placed under these adverse emergency conditions developed a leakage in the early postoperative course, far surpassing the insufficiency rates of lower GI sutures placed under elective conditions [20]. In fact, emergency surgery is a well-known independent risk factor for anastomotic leakage after colorectal surgery [20]. After elective colorectal cancer surgery, the reported incidence of anastomotic leakage ranges between 1 and 19%, with higher leakage rates after left colonic and rectal resections compared to right colonic resections [20]. In our patient collective 25% of the small to large bowel sutures and 27% of large to large bowel sutures developed a leakage as opposed to the reported insufficiency rates of 1–4% and 2–19%, respectively, under elective conditions [20, 31]. Most data concerning incidence and predisposing factors for lower GI suture leakage derive from elective colorectal surgery, leaving a marked paucity of information on the issue of primary suture placement during emergency laparotomy, with the exception of perforated diverticular disease. For perforated diverticulitis with purulent or fecal generalized peritonitis a series of randomized controlled trials (RCTs) triggered a shift in the indoctrinated non-restorative Hartmann approach by presenting primary anastomosis as a feasible alternative [17, 32–34]. In most of the mentioned RCTs, primary bowel reconstruction in the acute setting was accompanied by the placement of a diverting enterostomy by study design [32–34]. Only the LADIES trial allowed surgeons to decide whether or not to place a diverting enterostomy when performing primary reconstruction [17]. In our study, placement of a diverting stoma was defined as an exclusion criterium because of the high incidence of non-clinical (asymptomatic) leakage of distal sutures reported in the literature [35].

In an attempt to facilitate the choice of the appropriate surgical procedure in patients with generalized peritonitis due to perforated diverticulitis, a recent position paper defined septic shock, overall fitness to surgery and peritonitis severity as important factors to consider in the decision-making process [18]. While the notion of septic shock is clearly defined by the SEPSIS-3 consensus definitions, no explicit easy-to-use, “surgeon-friendly” scoring system for pre- or intraoperative assessment could be recommended based on current evidence. Immunocompetence, ASA-Score and MPI were suggested as adjutants in choosing restorative or non-restorative resections in hemodynamically stable patients [18].

As current guidelines and position papers ultimately leave the choice of the emergency operative procedure in the surgeons’ hands, the results of Karliczek et al. showing surgeons’ assessment to be a poor predictor for anastomotic leakage further consolidates the need of identifying objective criteria for selecting patients for primary bowel reconstruction under peritonitic conditions [36].

By the a priori exclusion of non-restorative resections, hemodynamic instable patients for which damage control surgery is the only obvious and valid option are not included in the present study. The preoperative ASA score did not discriminate between patients developing suture leakage and those who did not in our patient cohort. Neither did the intake of immunosuppressives or the chronic use of corticosteroids. The extent of peritonitis was, however, an independent predictor of suture outcome in the multivariate analysis. We chose to evaluate a simplified form of the MPI, developed to solely assess the extent and quality of the intraoperatively determined peritonitis for a number of reasons. First, the MPI was originally developed in 1987 for predicting postoperative morbidity in a cohort that also included peritonitis due to upper GI perforation but excluded postoperative peritonitis and mesenteric infarction [37]. Neither inclusion and exclusion criteria nor primary outcome matched the purpose of our study. Second, the MPI includes various parameters such as age, sex, preexisting malignancy that did not influence our primary outcome parameter in the univariate analysis. Third, it is easier for the operating surgeon to simply discriminate between purulent or fecal peritonitis and between localized or generalized peritonitis than to calculate a more intricate score. Our data show that patients with generalized fecal peritonitis developed a significantly higher rate of suture leakage (*p* =0.0014) than patients with less extensive peritonitis, while also showing a trend (*p* =0.0788) in the mortality rate.

The pathophysiological events triggered within the peritoneal cavity by the spillage of intestinal content seem to critically impact the complex and incompletely understood healing process of the sutured intestinal wall. Altered peripheral blood perfusion, bowel distention and intestinal wall edema are just few of the macroscopic changes imposing a greater degree of difficulty for the surgeon attempting primary bowel repair. The alterations on a microscopic and molecular level are just as intricate, as inflammatory status, microbiome and genetics all seem to affect intestinal suture healing [38]. In a histologic analysis of colonic tissue samples Stumpf et al. identified a preexisting impairment in collagen metabolism as a possible risk factor for the healing of enteric sutures [39]. Polymorphisms in lipid signaling and metabolic pathways are also thought to predispose to altered intestinal suture healing, underlining the importance of the preoperative patient status [38].

In our study, sCHE activity was the only relevant preoperative parameter identified as having a significant predictive value for suture outcome in the multivariate analysis. We deliberately chose to analyze sCHE activity instead of albumin in order to avoid data distortion by parenteral albumin infusions in patients that were hospitalized previous to emergency surgery. In support of sCHE as a predictor for anastomotic healing Antolovic et al. identified low preoperative sCHE levels as an independent risk factor for bile leakage in 519 patients who underwent hepaticojejunostomy [40]. In an emergency setting, our study is one of the few approaching the issue of preoperative predictors for a successful primary bowel reconstruction. Various studies have validated sCHE as a marker of nutritional status, correlating low sCHE levels to sarcopenia and to a high nutritional risk in critically ill patients treated on ICUs [26, 41, 42]. Beside the critically ill, oncologic patients are another group for which malnutrition importantly influenced postoperative morbidity and mortality [43, 44]. In patients with colorectal cancer, low sCHE levels were associated with poor 5-year overall and disease-specific survival rates [43], whereas nutritional support led to an increase in sCHE levels and in body weight [45]. In an analysis of 453 prospectively recruited treatment-naïve cancer patients, without manifest hepatic involvement, Pavo et al. reported that decreased sCHE is associated with an increased all-cause mortality [46]. Interestingly, an inverse correlation of sCHE with CRP was observed (r = − 0.21, *p* < 0.001) as in our study (r = − 0.40, *p* < 0.001). In another series of patients with non-malignant disease, sCHE was shown to negatively correlate with further parameters of inflammation, namely interleukin (IL)-6 and tumor necrosis factor alpha (TNF)-α [47].

The observed association with inflammatory parameters is not surprising since the body of evidence linking sCHE to the inflammatory response to injury is continuously growing. SCHE is part of the non-neuronal cholinergic system (NNCS), a complex regulatory network including most immune cells and regulating their function in the setting of local and systemic inflammation [48, 49]. By targeting this system through intraperitoneal injection of CHE inhibitors in an experimental abdominal sepsis model, Hofer et al. showed that locally administered CHE inhibitors led to a reduced production of pro-inflammatory cytokines and improved survival, most probably by increasing acetylcholine levels that control cytokine production [50]. This apparently beneficial effect of a lowered or inhibited CHE activity intuitively stands in contradiction with the clinical observation that a low sCHE activity measured at the clinical onset of sepsis is an independent predictor of worse outcome and higher mortality [51]. However, the anti-inflammatory effect of increased acetylcholine levels is expected to impair host defense against infections, which most probably offsets its benefit [52–54]. Several other studies on collectives of critically ill patients requiring ICU care identified low sCHE activity as a relevant predictor of increased mortality [27, 55, 56]. Peng et al. determined in an analysis of adult septic patients that every unit (kU/L) decrease in sCHE activity doubles the odds of death within 30 days from sepsis onset [27]. The exact mechanisms through which a reduction in sCHE activity leads to the observed results are far from being elucidated but suggest complex and intertwined derangements of metabolic and inflammatory pathways.

The two parameters, sCHE levels and the extent of peritonitis, put forward by our analysis to facilitate the intraoperative decision-making process during emergency surgery for secondary peritonitis require further prospective validation, as the retrospective and single center nature of the current study constitutes its major limitation. As discussed by previous authors, the recruitment of patients for RCTs in an emergency setting is challenging, as many patients are not able to give informed consent due to the severity of their condition. Several RCTs on emergency surgery for acute diverticulitis had to be prematurely terminated due to recruitment issues [32, 33]. Another shortcoming of the present study are the limited number of pre- and intraoperative variables considered for analysis. Although most of the standard laboratory parameters were accounted for in our study, further inflammatory and metabolic regulators that may affect postoperative wound healing need to be considered for a more accurate risk assessment. Nevertheless, the identified predictors of suture outcome have the benefit of being readily available at the time the decision on bowel reconstruction in SCS is due. A trial and error approach until having built one’s surgical experience is to be avoided at the expense of such a critical patient contingent. In the lack of data deriving from RCTs, the years of surgical experience compressed in the current study is a valuable stepping stone to further our understanding of intestinal suture healing in a peritonitic environment.

## Conclusion

Low preoperative sCHE activity and a high extent of the intraoperatively determined peritonitis are two easily quantifiable parameters that significantly correlate with a poor outcome of enteric sutures placed during SCS for secondary peritonitis. An objective surgical decision tailored to the patients’ individual pathophysiological pattern helps the surgeons, as they are no longer dependent on subjective considerations alone, while also benefiting the patients through the choice of the appropriate procedure.

## Data Availability

The datasets used and/or analysed during the current study are available from the corresponding author on reasonable request.

## Abbreviations

ASA: American Society of Anesthesiologists
BMI: Body mass index
CI: Confidence interval
CRP: C-reactive protein
CT: Computer tomography
ERAS®: Enhanced Recovery after Surgery
GI: Gastro-intestinal
Hb: Hemoglobin
ICUs: Intensive care units
IL: Interleukin
IQR: Interquartile range
MAP: Mean arterial pressure
MEWS: Modified Early Warning Score
MPI: Mannheim peritonitis index
NNCS: Non-neuronal cholinergic system
OR: Odds ratio
PCO_2_: Partial pressure of carbon dioxide
PO_2_: Partial pressure of oxygen
POD: Postoperative day
RCTs: Randomized controlled trials
sCHE: Serum choline esterase
SCS: Source control surgery
SIRS: Systemic Inflammatory Response Syndrome
SOFA: Sequential organ failure assessment
SSC: Surving Sepsis Campaign
SSI: Surgical site infection
TNF: Tumor necrosis factor
VIF: Variance inflation factors
WSES: World Society of Emergency Surgery
